# Letter from Prescott, Wis.

**Published:** 1881-12

**Authors:** 


### ARTICLE XI.

PRESCOTT, WIS., Sept. 29, 1881.

MESSRS. EDITORS :—I have recently had a case in which from personal considerations I was deeply interested, but it also possessed considerable professional interest to me, not having in thirty years' practice met a similar one, and also from the incertidude attending the diagnosis.

There are one or two points in the case on which I would like your opinion, and therefore inclose notes of the case. I shall be pleased to hear from you at your leisure.

Mrs H., aged twenty-six years, of highly nervous organization, general health rather feeble, has been married five years, and has suffered severely from dysmenorrhœa during that time, excepting last few periods. Supposed to be three months advanced in her first pregnancy. Soon after sitting on the damp ground, was attacked suddenly with severe pain in left side, near upper edge of iliac fossa, radiating in direction of distribution of upper lumbar nerves. There was considerable tenderness, with apparently an abnormal fullness in the region indicated, though no clearly defined tumor could be made out, the abdomen being well covered by adipose tissue. The pain was soon relieved by anodyne and warm applications. After the action of a cathartic, the fullness nearly but not entirely disappeared. During the next three weeks she had several similar attacks, lasting from a few moments to an hour or two; sometimes two or three in twenty-four hours, and then not for two or three days. At first there was no vesical irritation, but subsequently there was considerable vesical tenesmus present during the attacks of pain, which thus had the appearance of ordinary attacks of nephritic colic. The tenderness persisted, but was a source of but little annoyance. Finding that the attacks were much more apt to recur when on her feet, she remained most of the time in a recumbent position.

About three weeks after the first attack, Monday, four A. M., while asleep, she was taken with the same pain, but in an intensely aggravated form. I saw her within a few minutes, and found her screaming with agony. My first impression was that a rupture into the peritoneum of some abnormal growth—possibly a tubal pregnancy—had occurred; but the persistence of the pain, the severe vesical tenesmus present, and the absence of evidence of shock, except so much as might be fairly attributable to the pain, led me to conclude that a large and rough calculus was attempting the passage of the ureter. (Having recently, in my own person, had this experience, I had a vivid conception of the sufficiency of this cause for all the symptoms). She was as speedily as possible put under the influence of morphine, assisted at first by chloroform inhalations, this influence being kept up during the future of the case. The pain soon became bearable, but did not cease, as if the calculus had passed into the bladder, and I inferred that if a calculus, it had lodged in the ureter. Within thirty-six hours symptoms of severe general peritonitis had supervened, which, despite the treatment adopted, proved fatal Saturday eve, seven days from attack.

An autopsy (which unfortunately had to be conducted hastily) was held, which revealed the anticipated evidences of the fatal peritonitis. In the left iliac fossa was found the *origo mali*—a large, firm, round coagulum, including in its structure the ovary, Fallopian tube and broad ligament. The tumor was easily lifted from its bed, and was found to be at one point in contact with and attached to the left cornua of the uterus by a narrow, short pedicle, consisting of the ovarian ligament and Fallopian tube, twisted so as to have performed a complete revolution on itself. The broad ligament had within it two cysts filled with bloody serum, the larger the size of a large orange, the smaller half that size. The structures of the ovary, tube and broad ligament were so completely infiltrated with coagulated effused blood, that the whole mass of the tumor had the appearance of a homogeneous clot. Part of this coagulum within these organs, in appearance at least, seemed to antedate the main mass of coagulum. The point from which the hæmorrhage proceeded was not discovered. The tube itself was thought to be intact. Within the uterus was a four months fœtus.

The points of the case upon which I would like your opinion are: 1. The cause of the repeated attacks of pain. My own impression is that they were due to repeated small hæmorrhages which gradually infiltrated and distended the structure of these organs, until finally a rupture into the peritonial cavity occurred ; though, in this case, I do not see why an anodyne and warmth should have always controlled the pain within a short time.

2. Had the twisting of the pedicle (which I would have thought anatomically impossible without rupture of some of the tissues) anything to do with the attacks of pain or the hæmorrhage?

